# Development of the First Value Assessment Index System for Off-Label Use of Antineoplastic Agents in China: A Delphi Study

**DOI:** 10.3389/fphar.2020.00771

**Published:** 2020-06-16

**Authors:** Qian Jiang, Wei Zeng, Jiajie Yu, Hui Liu, Mian Mao, Youping Li

**Affiliations:** ^1^Chinese Evidence-Based Medicine Centre, West China Hospital, Sichuan University, Chengdu, China; ^2^Department of Pharmacy, Sichuan Cancer Hospital & Institute, Sichuan Cancer Centre, School of Medicine, University of Electronic Science and Technology of China, Chengdu, China; ^3^Department of Chronic Disease Prevention, Chengdu Center for Diseases Control and Prevention, Chengdu, China

**Keywords:** value assessment, index system, off-label use, antineoplastic agents, Delphi method

## Abstract

**Objective:**

To develop the first value assessment index system for off-label use of antineoplastic agents in China.

**Methods:**

A modified two-round Delphi method was employed to establish consensus within a ﬁeld to reach agreement *via* a questionnaire or doing interview among a multidisciplinary panel of experts by collecting their feedback to inform the next round, exchanging their individual knowledge, experience, and opinions anonymously, and resolving uncertainties.

**Results:**

Expert’s positive coefficient was 94.74% in the first round and 100.00% in the second round. In the first round, expert’s authority coefficient for a majority of 61 indicators was ≥ 0.80 (85.2%, ranging from 0.70 to 0.89, mean=0.84) and coefﬁcient of variation for all the 61 indicators was ≥ 22% (ranging from 11.67% to 21.74%, mean=17.4%). In two rounds, the mean expert’s authority coefficient raised to 0.85 (ranging from 0.75 to 0.90), and coefﬁcient of variation for all indicators was < 20% (ranging from 10.49% to 19.71%, mean=15.97%). The *P*-values of *Kendall’s W* test were all < 0.001 for each round. At the end of two rounds, *W*-value for concordance was 0.395 (χ^2^=347.494, *P*<0.0001). The final value assessment index system comprised of eight domains, 21 subdomains, and 56 indicators. The weight and combination weight of each domain were 0.4211 for therapeutic value, 0.1678 for source and type of evidence, 0.0961 for public feedback/comments, 0.0894 for novelty in drug discovery, 0.0689 for grading of evidence recommendation, 0.0578 for consistency of evidence results, 0.0561 for disease burden, and 0.0428 for ratio of composition/integration.

**Conclusion:**

Use of Delphi method to develop the proposed value assessment index system was found scientific and credible. This value assessment index system is highly appropriate for off-label use of antineoplastic agents in China.

## Introduction

The growing global cancer burden has accelerated the innovation in treatment, including the influx of new drugs. Nevertheless, skyrocketing healthcare costs, especially for antineoplastic agents combined with modest survival gains, raise questions that new anticancer drugs are not necessarily cost-effective (Kelly and Smith, 2014; Tefferi et al., 2015; Cohen, 2017).

Value, a relatively new, emerging and evolving term, which has eight separate but distinct definitions according to the *Oxford English Dictionary* (Oxford English Dictionary, ), is recognized as a multidimensional and dynamic concept with consensus, despite the fact that its definition may vary among different stakeholders, including physicians, payers, patients, *etc*. (Promoting Value, Affordability, and Innovation in Cancer Drug Treatment, 2018).

With promoting the use of high-value drugs, a number of organizations, including the American Society of Clinical Oncology (ASCO) (Schnipper et al., 2015; Schnipper et al., 2016), European Society for Medical Oncology (ESMO) (Cherny et al., 2015; Cherny et al., 2017), and National Comprehensive Cancer Network (NCCN) (National Comprehensive Cancer Network, ) have developed frameworks to assess antineoplastic agents either quantitatively or qualitatively, involving stakeholders (e.g., physicians, patients, and healthcare insurers). However, there is no a universally accepted framework and unfortunately no value assessment frameworks in developing countries, e.g. China, with scarce resources and rising demand for healthcare services.

Off-label use for antineoplastic agents, sometimes the only option for patients with advanced cancer in real-world settings, is an inevitable challenge and remains to be solved urgently in clinical practice. However, at present, lack of general specification and technical criteria for evaluation is tangible. Similar with “new drugs” or “new treatment” compared with a standard therapeutic regimen, it is feasible to use a value assessment framework to comprehensively evaluate off-label use for antineoplastic agents, so as to solve the technical bottleneck for evaluation of off-label use of antineoplastic agents.

Although there are compendiums of indicators for value assessment, there are currently no validated indicators to guide implementation and value evaluation of off-label use of antineoplastic agents. The aim of the present study was to explore establishment of the first value assessment index system for off-label use of antineoplastic agents in China using the modified Delphi method, which encompassed an iterative process and has been widely applied in diverse areas of healthcare system.

## Methods

**Figure 1:**
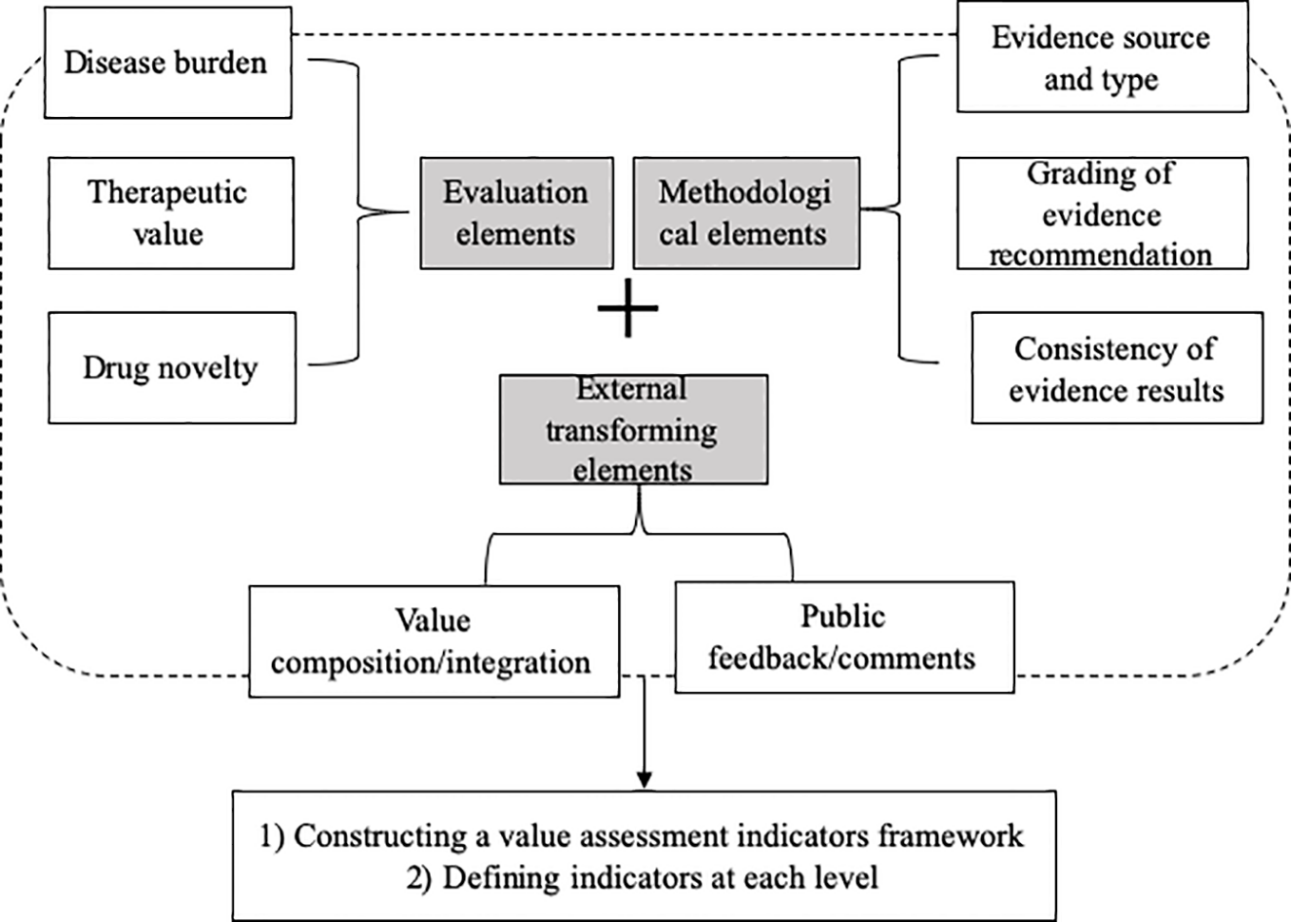
Study design of the Delphi method.

To develop a value assessment index system for off-label use of antineoplastic agents in China, a modified Delphi method was employed to establish consensus within a ﬁeld to reach agreement *via* a questionnaire or doing interview among a multidisciplinary panel of experts by collecting their feedback to inform the next round, exchanging their individual knowledge, experience and opinions anonymously, and resolving uncertainties (Hasson et al., 2000). Until consensus was reached on the final round, an agreement was identified (Iqbal and Pipon Young, 2009; Birko et al., 2015). The flowchart of the Delphi process is shown in **Figure 1**, and detailed description of the proposed system is presented in Appendix.

## Results

Totally, two rounds of consultation were carried out, and then, the consensus was reached.

### Characteristics of the Experts

A multidisciplinary panel enrolled 19 participants for consultation from geographically diverse areas, including North, South, and West of China who met experts’ defined criteria. Eighteen experts agreed to participate in the first and second rounds. All the experts had at least nine or more years of experience (range of experience, 9–29 years; mean, 18.2 years). Experts were predominantly (*n* = 17) working in hospitals and were employed in clinical pharmacy, pharmaceutical affairs, oncology, evidence-based medicine, clinical epidemiology and statistics, or pharmacoeconomics. Demographic characteristics of participants, including gender, profession, the highest level of education, etc. were also collected and shown in **Table 1**.

**Table 1:** Characteristics of expert panelists.

Categories	Characteristics	Number	Percentage (%)
Response to questionnaires	Round 1	18	94.7
	Round 2	18	100
Gender	Male	12	66.7
	Female	6	33.3
Educational attainment	Doctor’s degree	7	38.9
	Master’s degree	9	50
	Bachelor’s degree	2	11.1
Organization	Hospital	17	94.4
	Academic organization	1	5.6
Types of expertise	oncologists	4	22.2
	pharmacists	4	22.2
	Pharmacy director	5	27.8
	Policy makers	5	27.8
Main research areas	Clinical pharmacy	5	27.8
	Pharmaceutical affairs	5	27.8
	Oncology	4	22.2
	Evidence-based medicine	2	11.1
	Clinical epidemiology & statistics	1	5.6
	Pharmacoeconomics	1	5.6
Professional title	Senior	6	33.3
	Associate senior	12	66.7
Professional years	20~	6	33.3
	10–20	10	55.6
	<10	2	11.1
Province or region	Sichuan	12	66.7
	Chongqing	1	5.6
	Liaoning	1	5.6
	Guangdong	4	22.2
Familiarity degree	Very Familiar	2	11.1
	A little familiar	13	72.2
	Familiar commonly	3	16.7

### Expert’s Positive Coefficient

In general, expert positive coefficient (*C_aj_*) was 94.74% in the first round and 100.00% in the second round.

### Expert’s Authority Coefficient

In the first round, expert’s authority coefficient (*C_r_*) for majority of 61 indicators was ≥ 0.80 (85.2%, ranging from 0.70 to 0.89, mean=0.84), and coefﬁcient of variation (*CV*) for all the 61 indicators was ≥ 22% (ranging from 11.67% to 21.74%, mean=17.4%). After two rounds, *C_r_* for the majority of indicators was higher than that of the first round. The average *C_r_* raised to 0.85 (ranging from 0.75 to 0.90), and *CV* for all the indicators was < 20% (ranging from 10.49% to 19.71%, mean=15.97%), indicating that consensus has been achieved.

**Table 2** compares values of *C_r_* between the first round and the second round.

**Table 2:** Comparison of expert authority degree with two Delphi rounds.

Round one	Round two	Round one expert authority coefficient		Round one expert authority coefficient	
Candidate Indicators	Consensus on final indicators	Mean	CV (%)	Mean	CV (%)
Labor impairment extent	Reserve	0.83	18.14	0.85	10.61
Personal psychological burden	Reserve	0.81	16.6	0.81	13.29
Social activities extent	Reserve	0.76	19.9	0.75	16.19
Proportion of family expenses for illness	Reserve	0.84	15.24	0.84	12.69
Family members’ psychological burden	Reserve	0.79	17.66	0.81	14.43
Personal productivity affection on socio-economic	Reserve	0.77	18.4	0.79	13.67
Government image	Delete	0.73	19.4	–	–
popular psychology	Delete	0.7	20.93	–	–
Social stability	Delete	0.71	19.34	–	–
The necessity of off-label use for drugs	Reserve	0.89	11.67	0.88	11.81
Standard regimen available as control	Reserve	0.88	12.94	0.88	11.32
Hazard Ratio	Reserve	0.85	17.47	0.85	13.53
Overall survival rate	Reserve	0.86	17.54	0.87	12.8
Overall survival	Reserve	0.87	15.55	0.89	10.66
Progression-free survival rate	Reserve	0.87	17.02	0.87	13.51
Progression-free survival	Reserve	0.85	17.93	0.86	15.12
Overall response rate	Reserve	0.85	17.7	0.88	14.65
Adverse reaction grading	Reserve	0.89	12.65	0.9	10.49
Adverse reaction incidence	Reserve	0.89	12.35	0.9	10.78
Duration of adverse reactions	Reserve	0.88	13.65	0.88	12.39
Treatment-free interval	Reserve	0.81	19.17	0.82	18.26
Quality of life	Reserve	0.88	14.33	0.89	11.74
Symptoms remission with patients reported	Reserve	0.83	18.94	0.85	16.15
Treatment cost per course	Reserve	0.88	13.79	0.89	11.8
Proportion of patients’ out-of-pocket expenses (total course)	Reserve	0.87	13.83	0.88	12.28
Other forms of cost compensation	Reserve	0.8	21.74	0.77	25.31
Expenses proportion to develop and deploy new drugs	Delete	0.77	20.26	–	–
Development cycle for new drugs	Delete	0.77	20.69	–	–
Innovation international	Reserve	0.8	18.32	0.83	18.67
Innovation in China	Reserve	0.8	18.32	0.83	18.83
Affordable access for drugs (generic drugs or quality consistency evaluation of generic drugs)	Reserve	0.86	17.8	0.88	17.28
Therapeutic regimen alternative	Reserve	0.83	19.63	0.85	19.44
Clinical practice guideline	Reserve	0.88	18.52	0.88	18.18
Cochrane systematic review	Reserve	0.86	20.52	0.88	17.56
Other systematic reviews (including meta-analysis)	Reserve	0.86	18.53	0.86	18.69
Randomized controlled trial (phase III)	Reserve	0.89	18.76	0.88	19.1
Randomized controlled trial (phase II)	Reserve	0.87	17.94	0.86	18.77
Cohort study	Reserve	0.85	18.05	0.84	17.1
Case-control study	Reserve	0.83	18.86	0.83	17.97
Case series	Reserve	0.84	17.68	0.83	16.67
Case report	Reserve	0.86	17.11	0.85	16.75
Expert consensus	Reserve	0.87	16.12	0.87	16.08
Multidisciplinary collaboration	Reserve	0.87	15.55	0.86	16.37
Evidence submitted by pharmaceutical manufacturers	Reserve	0.84	16.71	0.82	16.76
Evidence recommendation	Reserve	0.86	18.71	0.87	18.56
Quality grading of evidence	Reserve	0.86	19.26	0.86	19.6
Validity	Reserve	0.85	17.24	0.86	17.54
Applicability	Reserve	0.85	18.16	0.85	19.02
Clinical importance	Reserve	0.86	17.62	0.87	17.36
Consistent results reported from at least two same type of study as evidence	Reserve	0.84	18.21	0.84	18.14
Single report as evidence	Reserve	0.83	19.08	0.83	18.27
New types of evidence	Reserve	0.84	17.74	0.82	17.85
Evidence updated	Reserve	0.85	18.05	0.84	18.59
Weight for indicators	Reserve	0.83	19.24	0.83	19.71
Weight for evidence type	Reserve	0.82	19.65	0.81	19.29
Weight for evidence grading	Reserve	0.81	19.52	0.83	16.85
Synthesis of evidence results	Reserve	0.79	16.59	0.78	14.99
Issued by association	Reserve	0.84	15.68	0.86	16.02
Issued by hospitals and its alliance	Reserve	0.85	13.26	0.86	13.73
Issued regularly	Reserve	0.86	16.15	0.85	16.14
Issued irregularly, as evidence updated	Reserve	0.86	14.72	0.85	15.23

### Degree of Coordination of Experts’ Opinions

*P*-values of Kendall’s *W* test were all < 0.001 for each round. At the end of the second round, *W*-value for concordance of final indicators was 0.395 (χ^2^=347.494, *P*<0.0001), which was statistically significant at the level of *α*=0.05, indicating that the consensus could be reached among the experts (**Table 3**).

**Table 3:** Result of expert opinion coordination degree.

Rounds	Round one			Round two		
Indicators	*W*-value	χ^2^	*P*-value	*W*-value	χ^2^	*P*-value
Domains	0.323	40.663	0.000	0.487	61.312	0.000
Subdomains	0.272	92.459	0.000	0.374	127.314	0.000
Indicators	0.310	289.800	0.000	0.395	347.495	0.000

### A Value Assessment Framework for Off-Label Use of Antineoplastic Agents

During the first round, the experts were invited to rate their opinions on 61 candidate indicators identified in the questionnaire. The second round was held to discuss answers from the ﬁrst round’s survey. After two rounds, a consensus of deleting five indicators, refining the expression of one indicator, and compressing indicators into three levels was achieved. Changes of indicators in two rounds were summarized in **Table 4**.

**Table 4:** Indicator changes in two Delphi rounds.

Original indicators	Modified results
Investment in drug research and development	Delete
Popular psychology	Delete
Government Image	Delete
Social stability	Delete
Cost ratio on research and development	Delete
Research and development cycle	Delete
Risk factors	Modified to: adverse effect

Consequently, after two rounds of consultation with experts, we generated an expert consensus around the final value assessment index system for off-label use of antineoplastic agents that was comprised of eight domains, 21 subdomains, and 56 indicators (**Table 5**).

**Table 5:** Value assessment index system.

Domains	Subdomains	Indicators	Combination weight
Disease burden	Individual burden	Abor impairment extent	0.85
		Personal psychological burden	0.78
		Social activities extent	0.66
	Family burden	Proportion of family expenses for illness	1.08
		Family members’ psychological burden	0.88
	Social burden	Personal productivity affection on socio-economic	1.36
Therapeutic value	Benefit value	The necessity of off-label use for drugs	1.43
		Standard regimen available as control	1.54
		Hazard ratio	1.45
		Overall survival rate	1.50
		Overall survival	1.50
		Progression-free survival rate	1.51
		Progression-free survival	1.49
		overall response rate	1.51
	Adverse reaction	Adverse reaction grading	3.58
		Adverse reaction incidence	3.33
		Duration of adverse reactions	3.14
	Survival value	Treatment-free interval	4.44
		Quality of life	4.44
		Symptoms remission with patients reported	4.22
	Economic affection	Treatment cost per course	2.56
		Proportion of patients’ out-of-pocket expenses (total course)	2.59
		Other forms of cost compensation	1.88
Drug novelty	Innovation value	Innovation international	2.18
		Innovation in China	2.14
	Alternative	Affordable access for drugs (generic drugs or quality consistency evaluation of generic drugs)	2.27
		Therapeutic regimen alternative	2.35
evidence source and type	Secondary studies	Clinical practice guideline	2.05
		Cochrane systematic review	1.95
		Other systematic reviews (including Meta-analysis)	1.78
	Clinical trials	Randomized controlled trial (phase III)	3.51
		Randomized controlled trial (phase II)	3.53
	Real world research	Cohort study	0.80
		Case-control study	0.79
		Case series	0.74
		Case report	0.71
	others	Expert consensus	0.33
		Multidisciplinary collaboration	0.32
		Evidence submitted by pharmaceutical manufacturers	0.28
Grading of evidence recommendation	Evidence evaluation methods/tools	Evidence recommendation	1.72
		Quality grading of evidence	1.71
	Evidence evaluation objective	Validity	1.12
		Applicability	1.12
		Clinical importance	1.21
Consistency of evidence results	Consistency	Consistent results reported from at least two same type of study as evidence	1.52
		Single report as evidence	1.37
	Update/correction	New types of evidence	1.47
		Evidence updated	1.42
Value composition/integration	Weight of results	Weight for indicators	0.70
		Weight for evidence type	0.65
		Weight for evidence grading	0.68
	Results forms	Synthesis of evidence results	2.25
Public feedback/comments	Public feedback mechanism	Issued by association	2.84
		Issued by hospitals and its alliance	2.53
	Frequency of public feedback	Issued regularly	2.16
		Issued irregularly, as evidence updated	2.08

## Discussion

### Main Findings

To our knowledge, this is the first Delphi-based study performed among a diverse panel of experts to develop a value assessment index system for off-label use of antineoplastic agents in China with eight domains, 21 subdomains, and 56 well-defined indicators at the end of two rounds. We believe that our study has filled an important gap on value assessment for off-label use of antineoplastic agents to address the difficulties in knowledge and practice in developing countries (e.g., China).

Although our framework was developed in the Chinese context, we believe that it can be implemented in other countries for assessing adherence to best decision-making, practice, and management in off-label use of antineoplastic agents.

### Strengths and Limitations

Our study has several strengths. Firstly, the eight domains in the framework, which were strongly endorsed by the experts, disease burden, and novelty in drug discovery, covered different types of cancer burden about the state, society, and individual, and also reflected the orientation of drug research and development policy in China. Source and type of evidence, grading of evidence recommendation, and consistency of evidence-based results highly condensed the methodological support of evidence-based medicine for the present study, and emphasized the importance of evidence and patients’ decision-making. These key domains were not proposed in other value assessment frameworks, and they therefore can be used in global scale.

Secondly, we invited 18 well-known experts in related fields who concentrated on clinical pharmacy, pharmaceutical affairs, oncology, evidence-based medicine, clinical epidemiology and statistics, and pharmacoeconomics, 16 of which had doctor’s or master’s degrees. The number of experts should be appropriately selected for a Delphi-based study (Iqbal and Pipon Young, 2009). Expert’s positive coefficient was 94.74% in the first round, while that was 100.00% in the second round, indicating that experts were interested in this research, and were willing to fill out the survey within the specified timeframe. The mean expert’s authority coefficient was 0.84 and that was ≥ 0.80 for the majority of 61 indicators in the first round, and then, raised to 0.85 in the second round, indicating a high degree of experts’ authority in the field of value evaluation of off-label use of antineoplastic agents in the Delphi surveys and qualifying them for participation in the survey.

Thirdly, a reasonable weight setting is crucial for establishing an index system. In the present study, therapeutic value plays a leading role in value assessment index system, demonstrating that multiple forms of evidence should be taken into account for value assessment, including but not limited to patient-reported outcomes, results from randomized controlled trials(RCTs), and real-world evidence as appropriate. The weight coefficient of the first-level indicators was in the following order: therapeutic value (0.4211), source and type of evidence (0.1678), public feedback/comments (0.0961), novelty in drug discovery (0.0894), grading of evidence recommendation (0.0689), consistency of evidence-based results (0.0578), disease burden (0.0561), and ratio of composition/integration (0.0428). Obviously, the top three weights were therapeutic value, source and type of evidence, and public feedback/comments, which highly condensed the methodological support of evidence-based medicine for the current study and emphasized the importance of evidence and patients’ decision-making (Wild et al., 2016).

Fourthly, open questions were raised during each round to gain more in-depth insight into the indicator, promoting more well-defined indicators, as well as ensuing guidance for satisfactory practice, so that the index system could be more appropriate for the purpose of value assessment.

However, the present study has also a number of limitations. Firstly, we did not include potential experts from some provinces or regions across China (i.e., east of China, which could limit our results). Secondly, we did not include payers of healthcare for consultation, which are important stakeholders in the value assessment of pharmacotherapy. Thirdly, the present study did not provide a face-to-face meeting for experts to discuss disagreement. Fourthly, Delphi consensus has its own limited validity. Fifthly, there were a great number of indicators, and it is therefore necessary to remove those indicators with low operability in the future according to empirical research on different types of cancer and drugs.

Future research should aim at setting an international consensus on a value assessment index system for off-label use of antineoplastic agents using Delphi method as a contribution to robust evidence for governments’ evidence-based decision-making, providing further insights into value and its relevance with drug prices to promote value-oriented medicine.

## Conclusions

We conducted a Delphi-based method and process to develop and validate the first value assessment index system for off-label use of antineoplastic agents in China. The ﬁnal 56 indicators need to be further tested, veriﬁed, and revised in clinical practice.

## Appendix

### Producing an Evidence-Based Candidate Indicators List

**Figure 2:**
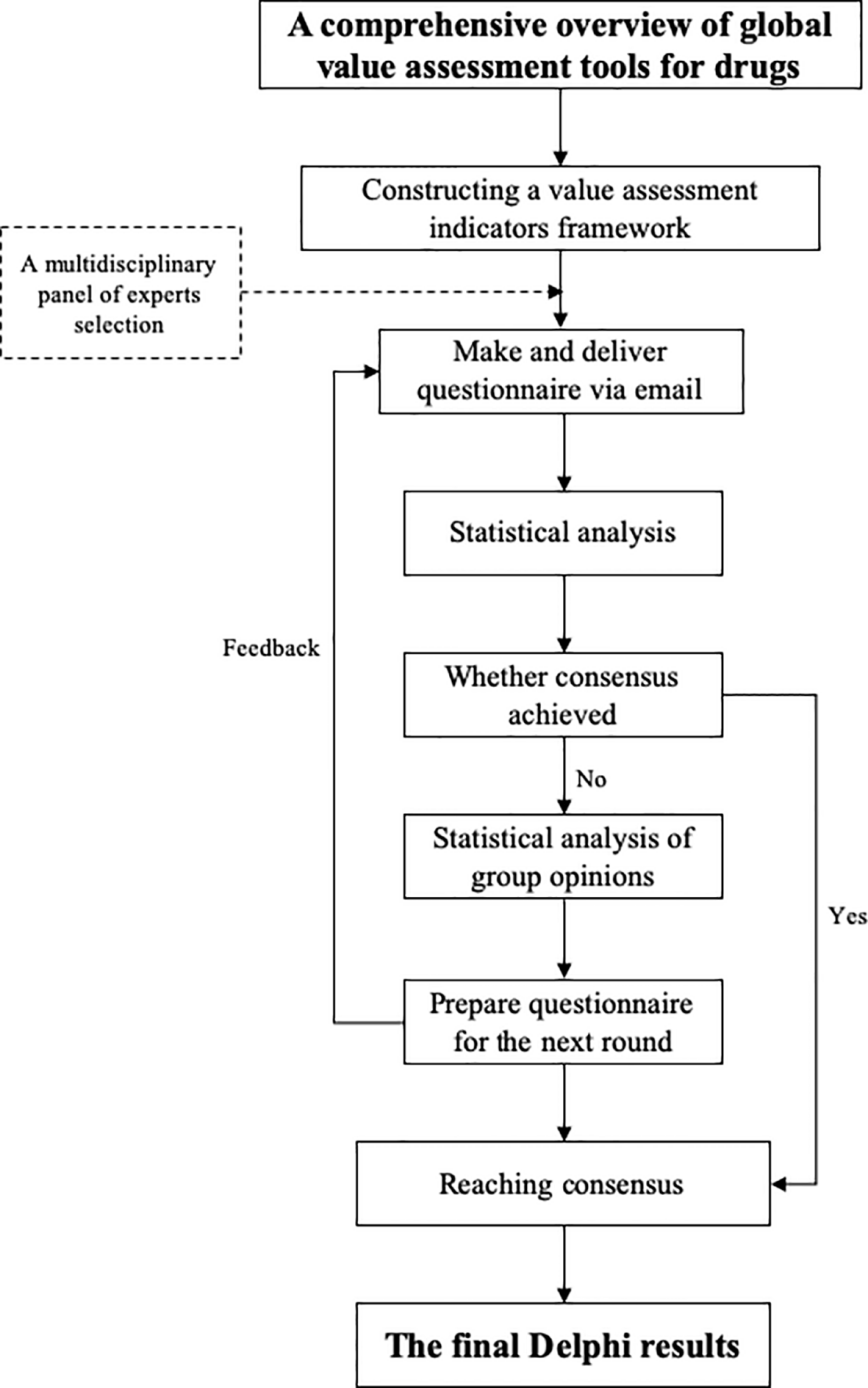
Conceptual framework of value assessment.

According to the comprehensive overview of global value assessment tools for drugs, a total of 12 eligible value assessment tools for drugs were identiﬁed (Jiang et al., 2019). They covered basic characteristics, key elements, and techniques in terms of value domains and metrics, evidence source/grading, development process, in which a detailed value assessment index system was presented and grouped into three levels, including eight domains, 22 subdomains, and 61 indicators. The eight domains were as follows: *i*) disease burden, *ii*) therapeutic value, *iii*) novelty in drug discovery, *i*v) source and type of evidence, *v*) grading of evidence recommendation, *vi*) consistency of evidence-based results, *vii*) ratio of composition/integration, and *viii*) public feedback/comments (**Figure 2**).

### Recruitment of Experts in a Multidisciplinary Panel

A purposive, criterion-based sampling approach was adopted to convene a multidisciplinary panel of experts, providing detailed explanation and objectives for our study to promote the acceptance of the final index system. With random sampling of mathematical statistics theory, the relationship between the mean sample standard deviation *σ* and the population standard deviation $\bar{\sigma }$ can be formulated as follows:

$$\bar{\sigma }=\frac{\sigma }{\sqrt{m}}$$

where, *m* represents the number of experts, and *m* increases, while $\bar{\sigma }$ decreases. Typically, a panel of 4~16 experts can bring out satisfying results, while for those relatively important issues, such as indicator design or weight distribution, 15~30 experts need to be considered to create diversity regarding representation (Akins et al., 2005; Sema and Rafa, 2012). Delphi does not use random sampling to recruit a panel of experts, in contrast to conventional surveys, which generally hold an aim of representativeness (von der Gracht, 2012; Khatwania and Karb, 2017).

### Materials and Consultation

Each round of questionnaire was delivered initially *via* e-mail and later by telephone. Experts were questioned about the importance, operability, and sensitivity, and then, ranked each indicator on a Likert-type scale (Akins et al., 2005) from 1-point (extremely inappropriate) to 5-point (extremely appropriate). Meanwhile, familiarity and judgment scores were recorded. Familiarity was divided into a Likert-type scale (Khatwania and Karb, 2017) where 1-point indicated that the expert is highly unfamiliar with the indicator and 5-point denoted that the expert is highly familiar with the indicator. Judgment criteria included four aspects: work experience, theoretical analysis, understanding from domestic and foreign counterparts, and intuition demonstrating the degree of influence, with scores of 1~3 points (**Supplementary Tables 1** and **2**). Besides, open questions through each round were allowed, so as to encourage experts to revise, delete or add indicators, which they perceived to be necessary for the index system prior to the following survey round. The indicators were adjusted and supplemented according to the experts’ comments. Then, the questionnaire was modified following the qualitative feedback and statistical analysis from the last round to the next. The final consultation with consensus achieved could lead to the final index system.

There is little evidence on the optimal number of Delphi rounds. Consensus is expected to increase with each additional round. However, potential bias also increases with experts’ fatigue or attrition. Therefore, mean scores ≥ 0.70 and *CV* ≤ 25% were set as the consensus level in the present study (Monguet et al., 2017).

### Statistical Analysis

The expert consultation database was established through Epi Data (version 3.1), exported into Excel 2016 spreadsheets, and all statistical analyses were carried out by SPSS 25.0 software (IBM, Armonk, NY, USA). Both descriptive statistics and quantitative analyses were undertaken. Each round of the Delphi survey was analyzed separately. A two-tailed *P*-value < 0.05 was considered statistically signiﬁcant. Descriptive information about experts’ gender, level of education, professional title, *etc*. was recorded.

The mean scores and *CV* were calculated for each indicator and round. *CV* ≤ 25% indicated less variability of the experts’ opinions.

Expert’s positivity coefficient (*C_aj_*) and expert’s authority coefficient (*C_r_*), involving two factors (the judgment criteria for the indicators (*C_a_*) and the experts’ familiarity with the indicators (*C_s_*)), were correlated together as follows: *C_r_*=(*C_a_*+*C_s_*)/2. *P*-values of Kendall’s *W* test and *W*-values were determined to evaluate the expert’s positive degree, expert’s authority degree, and degree of coordination of experts’ opinions (Khatwania and Karb, 2017). Mean scores ≥ 0.70 and *CV* ≤ 25% were recommended for Delphi studies and set as the consensus level (von der Gracht, 2012), and results that failed to meet either of the above-mentioned criteria indicated that no consensus could be achieved.

## Data Availability Statement

The raw data supporting the conclusions of this article will be made available by the authors, without undue reservation.

## Author Contributions

QJ: study design, drafting out and revising the manuscript critically for important intellectual content. WZ: study design, analysis and method directing. JY: participated in its design, revising the manuscript. HL: analysis and interpretation of data. MM: analysis and interpretation of data. YL: conceiving of the study, revising the manuscript finally.

## Conflict of Interest

The authors declare that the research was conducted in the absence of any commercial or financial relationships that could be construed as a potential conflict of interest.
