# Supplementary material for: Development of the First Value Assessment Index System for Off-Label Use of Antineoplastic Agents in China: A Delphi Study
Source: Front Pharmacol. 2020 Jun 16;11:771. doi: 10.3389/fphar.2020.00771 (PMC7308466; doi:10.3389/fphar.2020.00771)
Supplement: Supplementary file 1 [file DataSheet_1.doc]

Supplementary table 1: Degree of expert familiarity and a quantitative assignment of coefficient

| **Familiarity** | **Coefficient** |
| --- | --- |
| Very familiar | 1 |
| More familiar | 0.8 |
| Generally familiar | 0.6 |
| Less familiar | 0.4 |
| Unfamiliar | 0.2 |

Supplementary table 2: Judgment criteria and degree of influence

| **Judgment criteria** | **Degree of influence** | | |
| --- | --- | --- | --- |
|  | **High** | **Middle** | **Small** |
| Work experience | 0.5 | 0.4 | 0.3 |
| Theoretical analysis | 0.3 | 0.2 | 0.1 |
| understanding from domestic and foreign counterparts | 0.1 | 0.1 | 0.1 |
| Insight | 0.1 | 0.1 | 0.1 |
| **Total** | 1 | 0.8 | 0.6 |
